# Functional neuroimaging of the central autonomic network: recent developments and clinical implications

**DOI:** 10.1007/s10286-018-0577-0

**Published:** 2018-11-23

**Authors:** Miriam Sklerov, Eran Dayan, Nina Browner

**Affiliations:** 1 Department of Neurology, University of North Carolina, 170 Manning Drive, CB# 7025, Chapel Hill, NC 27599 USA; 2 Department of Radiology and Biomedical Research Imaging Center, University of North Carolina, 130 Mason Farm Road, CB# 7513, Chapel Hill, NC 27599 USA

**Keywords:** Central autonomic network, fMRI, Functional neuroimaging

## Abstract

**Purpose:**

The central autonomic network (CAN) is an intricate system of brainstem, subcortical, and cortical structures that play key roles in the function of the autonomic nervous system. Prior to the advent of functional neuroimaging, in vivo studies of the human CAN were limited. The purpose of this review is to highlight the contribution of functional neuroimaging, specifically functional magnetic resonance imaging (fMRI), to the study of the CAN, and to discuss recent advances in this area. Additionally, we aim to emphasize exciting areas for future research.

**Methods:**

We reviewed the existing literature in functional neuroimaging of the CAN. Here, we focus on fMRI research conducted in healthy human subjects, as well as research that has been done in disease states, to understand CAN function. To minimize confounding, papers examining CAN function in the context of cognition, emotion, pain, and affective disorders were excluded.

**Results:**

fMRI has led to significant advances in the understanding of human CAN function. The CAN is composed of widespread brainstem and forebrain structures that are intricately connected and play key roles in reflexive and modulatory control of autonomic function.

**Conclusions:**

fMRI technology has contributed extensively to current knowledge of CAN function. It holds promise to serve as a biomarker in disease states. With ongoing advancements in fMRI technology, there is great opportunity and need for future research involving the CAN.

## Introduction

**Fig. 1:**
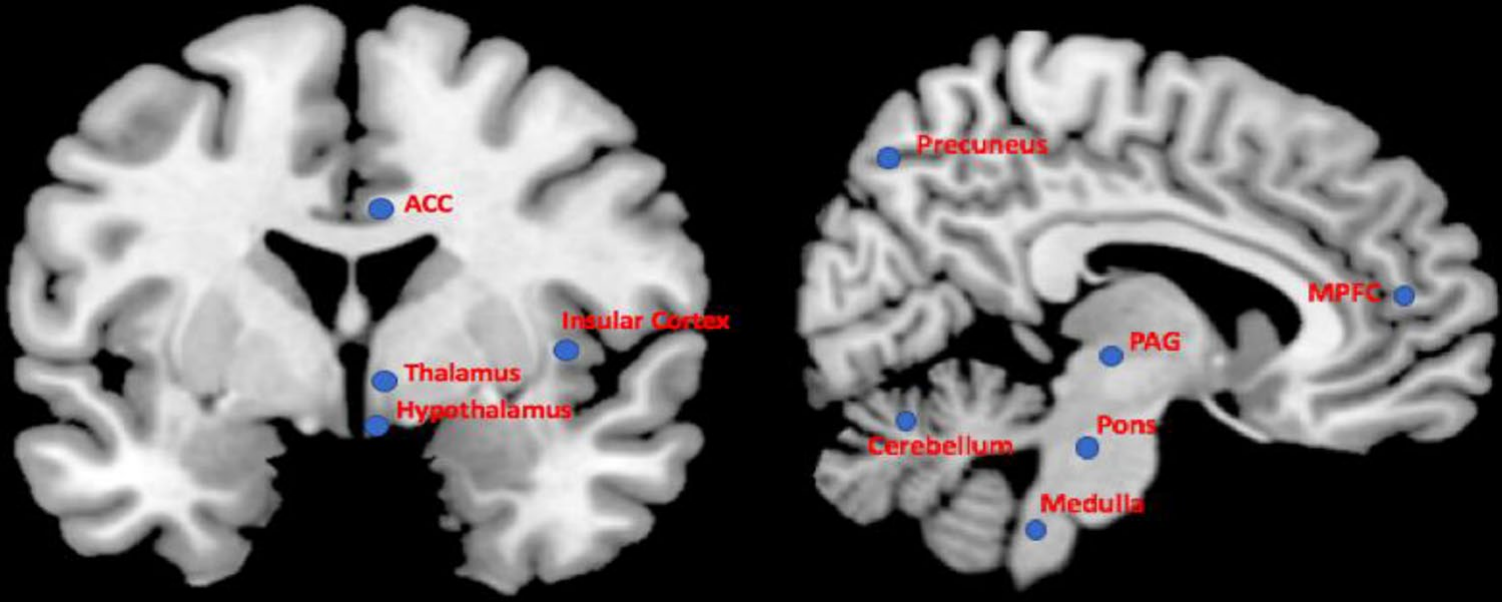
Brain structures currently implicated in the CAN. Not shown: Amygdala, hippocampus. *ACC* anterior cingulate cortex, *MPFC* medial prefrontal cortex, *PAG* periaqueductal gray

The central autonomic network (CAN) is an intricate network of brainstem and forebrain regions that are implicated in both baseline autonomic nervous system (ANS) function, as well as the modulation of ANS function in response to changing environments. The CAN has been studied extensively in animals, and via lesional and pathologic studies in humans [1]. The most highly implicated brain regions from these studies have been the insula, amygdala, hypothalamus, periaqueductal gray (PAG), parabrachial complex, nucleus of the solitary tract (NTS), and the ventrolateral portions of the medulla [1] (Fig. 1); thus, these regions were classically considered to be the regions that made up the CAN.

From a functional and methodological perspective, the CAN can be divided into three hierarchical levels. The spinal cord contains neuronal bodies and projections which direct segmental reflexive control of ANS function. At the level of the brainstem, the NTS, the ventrolateral medulla (VLM), and parabrachial nucleus of the dorsolateral pons are implicated in immediate and reflexive control of circulation, respiration, gastrointestinal function, and micturition. The PAG in the midbrain region integrates autonomic control with pain modulation and behavioral responses to stress and sleep. Finally, at the level of the forebrain, the hypothalamus integrates autonomic, endocrine, and sleep functions, and the anterior limbic circuit [anterior cingulate cortex (ACC), amygdala, and insular cortices] integrates bodily sensation and pain with emotional and goal-related autonomic responses [1]. Forebrain structures are implicated in modulation of ANS reflexive functions in response to internal and external environments.

Prior to the incorporation of functional neuroimaging, in vivo research into the CAN function in humans was rather limited. Functional neuroimaging has improved our understanding of the CAN function. It has also helped to identify important CAN brain structures which had not previously been strongly implicated in human ANS function, such as the thalamus, medial prefrontal cortex (mPFC), precuneus, cerebellum, and ACC (Fig. 1). In this review, we will describe work which has led to improved understanding of the CAN using functional neuroimaging, specifically functional magnetic resonance imaging (fMRI). We will begin with a discussion of functional neuroimaging, particularly fMRI, and its application in CAN research, including the strengths and limitations for its use in this area, and recent advances which will improve research using this modality. We will then describe fMRI studies of normal CAN function which have been conducted in healthy human subjects, with a focus on methodologies that have been utilized to investigate the CAN specifically. We aim particularly to highlight important advances in this area. This will lead us into the discussion of fMRI research geared at understanding ANS dysfunction in disease states. We will conclude by stressing areas for future research.

As many of the structures within the CAN play important roles in other central networks, particularly emotional, nociceptive, and cognitive pathways, we have kept the focus of this review on studies directly testing autonomic networks and tasks, with occasional exceptions made to highlight specific points. Scientific papers relating to pain [2], emotion [3], cognitive function [4], and affective disorders are out of the scope of this review, though there has been exceptional work in functional neuroimaging relating to CAN structures within this literature. In fact, some have postulated that rather than representing a static network, the CAN may symbolize a series of functions which are “borrowed” from other networks in response to a changing environment [5]. Similarly, work on functional neuroimaging of vagal nerve stimulation with relation to treatment of depression, epilepsy, pain, and effects on behavior will not be discussed here with few exceptions made to highlight the brainstem autonomic projections.

## Functional neuroimaging of the central autonomic network: technique, strengths, and considerations

Multiple imaging modalities have been used for studying different aspects of ANS function and dysfunction. Magnetic resonance imaging (MRI) modalities such as structural and diffusion MRI, for example, were used for delineating macro and microstructural properties of gray (e.g., [6]) and white matter (e.g., [7]) associated with autonomic functions, or altered in disease states that implicate the ANS. Other metabolic and molecular imaging modalities, such as positron emission tomography (PET), were used for studying the underlying physiological mechanisms of central and peripheral ANS function, and the involvement of various neurotransmitter systems (e.g., [8–10]). While studies based on these imaging modalities contributed invaluable information on ANS function and dysfunction, fMRI has provided the largest body of research in this area, mostly due to its non-invasive administration, high spatial resolution, and reasonably high temporal resolution. Therefore, for clarity and brevity, our focus here will be solely on studies that utilized fMRI.

With its relatively high spatial resolution, non-invasive application and ability to allow simultaneous whole-brain data acquisition, fMRI has played a major role in CAN research in the past 25 years. The vast majority of fMRI studies are based on the blood-oxygen-level dependent (BOLD) signal, which is thought to reflect local neural activity-dependent changes in the relative concentration of oxygenated and deoxygenated blood [11]. As in relation to other behavioral, cognitive, and affective functions, fMRI studies that focused on the ANS utilized both task-based and task-free designs. In task-based designs, BOLD signals are acquired while subjects are engaged in tasks, the execution of which is time-locked to image acquisition. A range of statistical approaches can then be used to identify associations between task performance and regional brain activity. More recently, and with increasing popularity, studies have also been utilizing task-free fMRI, where BOLD signals are acquired at rest, while subjects are not engaged in any particular task. In this approach, commonly referred to as resting-state fMRI (rsfMRI), the statistical dependency (“functional connectivity”) between BOLD signals derived from anatomically distinct brain regions is calculated [12, 13], and is postulated to reflect intrinsic interactions within large-scale brain systems. Functional connectivity measures can be compared among patients and controls [14], or can be used to evaluate the effects of pharmacological or other clinical interventions.

**Fig. 2:**
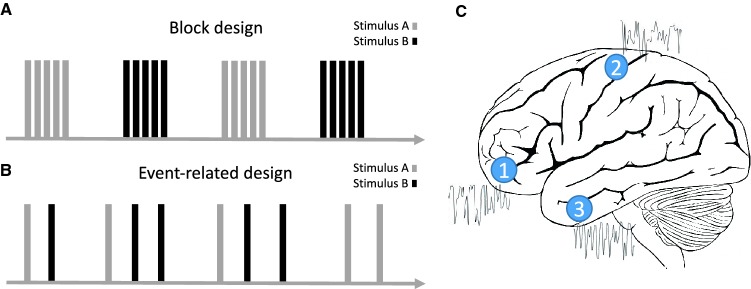
Illustration of common functional MRI protocols. **a** Block-design task-based protocol. **b** Event-related task-based protocol. **c** Resting-state protocol. See text for a detailed description of each type of protocol

Task-based fMRI designs have been instrumental in CAN research. Such designs can be generally divided into two forms, both of which are widely used in ANS research. First (Fig. 2a), in block-design paradigms, experimental conditions are presented continuously in a ‘blocked’ manner, for a set amount of time. Blocked presentations typically alternate with one another, separated by periods of ‘rest’. For example, the breath-holding task [15], used for studying vascular reactivity is typically administered in a block design, with alternating blocks of breath-holding and recovery. In a second common fMRI design (Fig. 2b) BOLD responses to more discrete and brief events are studied. One example for these so-called *event*-*related* designs is the volitional swallowing task [16], where discrete swallowing events are studied and analyzed. While block designs are typically more robust, event-related designs allow randomizing trial presentations, and more directly examine temporally dissociable components of trials (e.g., response preparation, response to feedback, etc.) [17]. More generally, these designs allow for more flexibility in the studied paradigm [18, 19].

Autonomic tasks are commonly used in physiologic studies of the ANS, and are easily replicated in the MRI scanner. Performance of baroreflex-activating or de-activating tasks, such as Valsalva, cold exposure, neck suction, static handgrip exercise, and lower body negative pressure (LBNP) have aided in the study of CAN brainstem and forebrain structures [20–23] (Table 1). The Valsalva maneuver, which is performed by asking the subject to exhale forcefully against resistance, causes a reduction in venous return and subsequent fall in systolic blood pressure, which triggers activation of the sympathetic nervous system primarily via the baroreceptor arc. This results in an initial brief drop in blood pressure, followed by an increase in heart rate, cardiac contractility, and blood pressure. Cold exposure activates specific sympathetic pathways that result in cutaneous vasoconstriction to protect the body’s core temperature [24]. Neck suction maneuvers cause an increase in transmural carotid pressure which simulates a drop in mean arterial blood pressure, while LBNP mimics an orthostatic blood pressure reduction, both leading to baroreflex activation. Investigations using muscle exertion, such as the static hand grip exercise, and exploring the mechanism of muscle sympathetic nerve activity (MSNA) have provided further insights into the brainstem baroreflex physiology in humans [25–28]. Ergoreceptors in the muscle sense increased activity levels, and signal increases in blood delivery to muscles and skin, in large part by increasing heart rate [29, 30]. Physiologic measurement of MSNA via electrodes in the muscle are thought to provide a direct measurement of peripheral vasoconstrictor tone, which is exclusively sympathetically mediated. MSNA is time locked with the cardiac cycle via the baroreflex arc [26, 29]. When baroreceptors are unloaded, i.e., during diastole, MSNA activity increases [29].

The acquisition of reliable signals from key CAN structures, for example from the brainstem and spinal cord, is challenging, given their proximity to fluid (CSF) and major arteries, which contaminate the BOLD signal with non-related physiological noise [31, 32]. Overcoming these inherent obstacles in the brainstem has been achieved by the introduction of cardiac (or respiratory)-gating, wherein BOLD image acquisition is time-locked to a specific point in the cardiac or respiratory cycle [31], effectivity minimizing the contaminating effect of physiological noise. Other methods that rely on pre-processing strategies have also been developed and routinely applied [31]. Functional neuroimaging in the spinal cord remains extremely limited.

## Functional MRI of the central autonomic network in healthy adults

Many methods have been employed to delineate the architecture and function of the CAN in healthy humans. Numerous studies have employed physiological measures, such as heart rate variability (HRV) and skin and muscle sympathetic responses, in conjunction with functional neuroimaging to associate activation of CAN structures with measurements of autonomic function. Task-based designs have been employed to induce functional engagement of CAN structures (Table 1). Autonomic tasks that are used to study the CAN include Valsalva, breath holding, static handgrip, application of cold, and negative lower body pressure [33]. The use of more complicated tasks with fMRI, such as administration of emotional or painful stimuli, cognitive exercises, and nauseogenic visual stimuli have also been employed to try to better understand the intricate functions of, and interactions within, the CAN [2–4, 34]. More recently, rs-fMRI has been a very useful tool in in vivo studies of CAN structures. Together, these methods have provided invaluable data about the CAN. In this section, we will first describe fMRI studies and advances focused on the brainstem, followed by those focusing on forebrain regions. The techniques used to study these areas will be emphasized.

### Brainstem

Brainstem CAN structures involved in ANS function have significant roles in beat-to-beat regulation of the cardiovascular system, respiration, gastrointestinal function, and micturition, which have been demonstrated in animal models [35–39]. Neuroimaging of brainstem structures in humans has proven difficult for several reasons, relating to inherent qualities of the brainstem. Namely, close proximity to large blood vessels and ventricles, as well as the small size and poor demarcation of brainstem nuclei, have made this area difficult to image with MRI techniques [40]. Image acquisition and analysis requires high resolution and careful, high quality analytic techniques.

#### Brainstem fMRI during autonomic tasks in healthy subjects

Through recent advances in functional neuroimaging, in vivo human studies have supported the hypotheses that much of the distribution of autonomic function in brainstem structures is conserved from lower mammals to humans [41], and expanded our understanding of the circuitry involved in CAN function. Earlier studies began to demonstrate the utility of fMRI in investigation of brainstem structures of the CAN by examining responses to the Valsalva maneuver and other ANS challenges [20–22, 42]. Pressor challenges, such as Valsalva and cold application to hand or forehead, elicited responses in the midbrain and pontine regions, as well as distributed forebrain autonomic centers [20, 23]. The expected physiologic response in heart rate and blood pressure were found to correlate well with fMRI signal change [22, 23]. Though spatial resolution in the brainstem regions is limited, areas of activation during tasks that activate the general visceral autonomic system correlate to known brainstem CAN structures involved in sympathetic modulation and outflow, such as the NTS, parabrachial complex, and PAG [42].

Collection of fMRI data during performance of pressor challenges has demonstrated not only the distribution of activity in the brainstem, but also the time course of activation of CAN structures during these tasks and their correlation with physiologic changes in heart rate [22, 42]. Activation was seen in the dorsal medulla, the midline medulla, the dorsal pons (felt to include the parabrachial complex), and the dorsal and ventral midbrain encompassing the PAG [22, 42]. It was noted that the time course of Valsalva-induced brain activation mirrored the measured heart rate response during Valsalva, which further supports the role of these structures in increasing sympathetic activity in response to this pressor challenge [22].

The brainstem involvement in the control of MSNA in humans was hypothesized, but not confirmed prior to contributions from functional neuroimaging. Animal studies as well as physiologic observations in humans had been used to investigate this pathway, leading to a putative mechanism primarily involving the NTS and its projections to other brainstem structures, with outflow through the rostral VLM [43–45]. To study this mechanism in humans, several approaches have been employed. Sustained hand-grip tasks correlated with activation in the medial and lateral dorsal medulla, believed to correspond to the NTS and rostral VLM, further implicating the involvement of these structures in the MSNA arc in humans [25, 46]. With the advent of MRI-compatible electrodes, simultaneous MSNA and fMRI data collection was made possible [21, 26–28]. Using a tungsten microelectrode in the common peroneal nerve, Macefield et al. recorded MSNA at rest in healthy adults while undergoing fMRI [28]. They observed coupling of nerve signal intensity with BOLD signal intensity in structures within the medulla. Specifically, increases in intensity were observed in the area of the rostral VLM, while decreases were seen in the regions corresponding to the NTS and caudal VLM, again implicating these regions in regulation of MSNA [28]. A maximal inspiratory capacity apnea (maximal inspiratory breath-hold) can be used to evoke an increase in MSNA [47]. In association with increased MSNA as measured via electrode recordings from the common peroneal nerve, while controlling for global alterations of BOLD signal attributable to hypercapnia, the inspiratory capacity task produced changes in fMRI signal intensity in the area of the rostral VLM, regions encompassing the NTS, and the dorsomedial hypothalamus [21]. Collectively, these studies support a sympathetic activation role of the rostral lateral medulla, and likely a sympathetic inhibitory effect of the caudal lateral medulla in the MSNA reflex. Taken together, brainstem fMRI studies utilizing pressor challenges provide key evidence of the brainstem structures involved in the human baroreflex arc, which had previously been suspected through animal studies and observational studies in humans.

#### Brainstem fMRI in direct vagal nerve stimulation in healthy subjects

Direct stimulation of the vagus nerve with concurrent fMRI has been useful in delineating vagal structures in the human brainstem [48, 49]. With application of external electrical stimulation of the neck overlying the vagus nerve, Frangos et al. observed activation of classic vagal afferent projections, including the NTS, parabrachial area, primary sensory areas, and the insula [49]. External stimulation of the auricular branch of the vagus nerve, which had previously not been shown to send projections to brainstem vagal areas, also demonstrated activation of the NTS, hypoglossal nucleus, locus coeruleus, parabrachial area, and PAG [48]. fMRI in these studies was able to provide in vivo evidence of brainstem vagal nerve projections in humans to confirm previous animal, physiological, and post-mortem studies.

#### Brainstem resting state fMRI in healthy subjects

Rs-fMRI, or task-free fMRI, has also emerged as an important tool in understanding the CAN. One major advantage of rs-fMRI is that the subject is not asked to perform a complicated task, minimizing the contribution of non-specific factors contributing to task success such as motivation, attention, or anxiety. This may be particularly important as many CAN structures overlap with other brain networks, such as those controlling emotional, cognitive, and sensory functions. Based on correlations with HRV measurements during rs-fMRI, the NTS and parabrachial pons were identified as important brainstem structures in sympathetic and parasympathetic function [50]. Measurement of MSNA at wakeful rest has provided similar advantages over task-based fMRI studies [26–28].

### Forebrain

Classically, forebrain structures of the CAN include the hypothalamus, insula, and amygdala [1]. These structures are key in both bottom-up relay of autonomic inputs, as well as top-down regulation and modulation of the autonomic responses, and integration of painful, cognitive, emotional, sensory, endocrine, and sleep related information into autonomic functions [1].

Functional neuroimaging focusing on forebrain ANS structures has provided many important insights into the human CAN. This work has revealed that in addition to brainstem structures, higher level cortical structures and intricate interactions within these structures in humans are involved in “reflexive” autonomic function. Additionally, structures that had previously had an undefined role in the human CAN, such as the mPFC, thalamus, ACC, hippocampus, precuneus, and cerebellum have been identified as key players in important ANS functions. Specificity of autonomic function, particularly lateralization of cortical autonomic functions, and specificity of subregions of larger brain structures, has been discovered using fMRI. Finally, studies using functional neuroimaging have allowed a better understanding of mechanisms of modulation of ANS control.

#### Cortical fMRI during autonomic tasks in healthy subjects

The cortical responses to baroreceptor activation had been suspected from animal studies. Task-based imaging designs were first employed to study this reflex arc. Tasks that have effects on sympathetic outflow via the baroreflex arc, such as Valsalva, maximal inspiration, isometric hand grip, and cold application to the forehead in conjunction with fMRI were particularly useful in charting related cortical activity [20, 22, 51, 52]. These studies demonstrated activation of the orbitofrontal cortex encompassing the ACC, temporal cortex, insular cortex, hippocampal and amygdala areas, thalamus, and hypothalamus in association with maximal inspiration, Valsalva, isometric handgrip tasks, and/or cold application [20, 52]. Activation of the insula in particular appears to follow the time course of heart rate and brainstem responses to the Valsalva maneuver [22], highly implicating this region in the sympathetic outflow response to the maneuver. Additionally, different gyri of the insula respond preferentially to different stimuli [51], indicating specificity of substructures within the insular cortex. Studies using LBNP, as a means of providing a direct hypovolemic stimulus, also induced activation in the right posterior insula, as well as the left cerebellar hemisphere and areas of the frontal and parietal cortices which correlated with measured increases in heart rate during the stimulus [53]. LBNP elicited reduced activity in the bilateral anterior insular cortices, right ACC, amygdala, midbrain and mediodorsal thalamus [53]. Interestingly, physical deconditioning, as produced by head down bed rest (HDBR), which results in a state of hypovolemia, specifically alters the activity of the ACC in response to LBNP when compared to diuretic-induced hypovolemia, correlating with the enhanced heart rate response to LBPN [54]. Similarly, post-exercise heart rate responses to LBNP is augmented, which correlates with larger increases in BOLD signal within the insula and ACC in response to LBNP, as well as greater decreases in the thalamus and mPFC, implicating a modulatory effect of these structures on baroreceptor sensitivity and function [55]. Taken together, the insula and ACC are cortical structures consistently implicated in increased sympathetic outflow in response to tasks that activate the baroreflex arc. The roles of other cortical regions, particularly the mPFC, thalamus, and amygdala, require additional investigation.

Maximal inspiratory capacity and breath holding maneuvers were shown to induce activation of the insular cortex, ACC, and the cerebellar cortex in a pattern associated with timing of increased MSNA in response to these maneuvers [56], similar to what is seen with Valsalva and LBNP. Maximal inspiratory capacity with concurrent measurement of MSNA also reveals activation in the anterior insula, dorsomedial hypothalamus, and ACC, as well as in the brainstem and deep cerebellar nuclei described previously [21]. Reduction in signal intensity was seen in the hippocampus, cerebellar and posterior cingulate cortexes, correlating with reduced activity in medullary structures [21]. Cortical regions, particularly the insula, the ACC, and the mPFC, appear to play both afferent and efferent roles in the MSNA reflex as evidenced with fMRI [25, 57].

**Table 1:** Brain regions with altered fMRI signal during autonomic tasks

Autonomic task	Brain regions demonstrating change in fMRI signal	Activation/deactivation
Valsalva [20, 22, 23, 51, 52, 72]	Medial prefrontal cortex	+
	Temporal cortex	+
	Amygdala	+
	Hippocampus	+
	Thalamus	+
	Hypothalamus	+
	Cerebellum	+
	Pons	+
	Medulla	+
	Midbrain	+
	Insula	+
Cold application [20, 23]	Orbital/medial prefrontal cortex	+
	Temporal cortex	+
	Amygdala	+
	Hippocampus	+
	Thalamus	+
	Hypothalamus	+
	Cerebellum	+
	Pons	+
	Medulla	+
	Anterior insula	+
Inspiratory capacity apnea, breath-holding, and Mueller’s maneuver [21, 52, 56]	Rostral lateral medulla	+
	Cerebellum	±
	Insula	+
	Hypothalamus	+
	Anterior cingulate cortex	+
	Thalamus	+
	Medial prefrontal cortex	+
	Dorsomedial and caudal lateral	−
	Medulla	−
	Hippocampus	−
	Posterior cingulate cortex	−
Static handgrip [25, 46, 51, 52, 57, 67, 68, 71]	Medial medulla	+
	Dorsolateral medulla	+
	Anterior insula	+
	Thalamus	+
	Medial prefrontal cortex	±
	Posterior insula	+
	Subgenual (ventral) anterior cingulate cortex	+
	Medial cingulate cortex	+
	Posterior cingulate cortex	+
	Putamen	+
	Cerebellum	+
	Hippocampus	+
	Dorsal anterior cingulate cortex	±
		−
Lower body negative pressure [53–55, 74]	Insula	±
	Cerebellum	+
	Frontal cortex	+
	Parietal cortex	+
	Genual/dorsal anterior cingulate cortex	+
	Sub-genual (ventral) anterior Cingulate cortex	−
	Amygdala	−
	Midbrain	−
	Thalamus	−
	Ventral medial prefrontal cortex	−

+ increased activity, − decreased activity, ± activity is increased in some studies and decreased in others

#### Resting-state fMRI of forebrain structures in healthy subjects

MSNA at rest provides an excellent approximation of baroreflex unloading during diastole. MSNA bursts were correlated with increased fMRI BOLD signal intensities in the precuneus, posterior cingulate cortex, left hypothalamus, dorsolateral prefrontal cortex, and the deep cerebellar nuclei [26, 58]. Analysis of HRV in conjunction with functional neuroimaging has revealed more complicated interactions of many brain regions in the CAN [59], particularly the mPFC and ACC, the ventral striatum, and the amygdala. Differentiating sympathetic and parasympathetic drivers of HRV in the CAN has been attempted, where high frequency (HF) HRV is generally accepted to reflect parasympathetic (vagal) outflow to the heart while low frequency (LF) HRV have been postulated to reflect cardiac sympathetic and parasympathetic activity [60], though this delineation remains controversial. Concurrent HRV measurement and fMRI was first accomplished by Critchley et al., demonstrating ACC activation in conjunction with sympathetic activity at rest [61]. HRV analysis has also been used to identify activation of the ACC [62], amygdala and cerebellum [63] in conjunction with parasympathetic cardiovascular activity. While focusing on functional connectivity of the dorsal ACC and the amygdala, Chang et al. [64] demonstrated that high frequency HRV correlated with increased functional connectivity among seed regions and the brainstem, thalamus, and hypothalamus. Meanwhile, low frequency HRV correlated with functional connectivity between both seeds and the parieto-occipital cortex. Though these results are intriguing, variability in independent variable measurement (R–R interval, HR analysis, or HRV) remains a challenge when comparing studies [65].

#### Functional specificity of cortical CAN structures

The advent of functional neuroimaging has allowed for confirmation of the involvement and function of previously implicated CAN structures and discovery of involvement of previously unanticipated regions. Additionally, functional neuroimaging has led to the discovery of the specificity of substructures in these regions, the lateralization of the function of CAN structures, and even gender-specific variations in CAN function.

Recent meta-analyses have revealed consistent and significant roles of the anterior and midcingulate cortices, insula, ventromedial prefrontal cortex, mediodorsal thalamus, amygdala, hippocampus, and hypothalamus in the CAN [5, 59]. The use of fMRI has also allowed further probing into the specific autonomic functions of these structures. De-activation of the mPFC in concordance with increasing heart rate has implicated the role of this region in efferent vagal activity [66]. The strong negative correlation between mPFC activity and heart rate has been demonstrated in additional studies during hand grip exercises [57, 67, 68]. Analysis of functional connectivity of the mPFC has uncovered greater HRV at rest correlations with stronger functional connectivity of the mPFC with the amygdala [69], and has revealed functional connectivity between the mPFC and other structures of the CAN, particularly the amygdala and the hippocampus [67, 69]. Parcellation of the insula for fMRI has shown differential functions in this region, with preferential responses to different autonomic challenges in each area, specifically implicating the anterior insula in sympathetic responses [51]. Similarly, parcellation of the hypothalamus and orbitofrontal cortex has revealed specificity for functional connectivity between the medial hypothalamus (which is involved in cardiovascular regulation) and the medial orbitofrontal cortex, implicating the orbitofrontal cortex in regulation of cardiovascular autonomic control in humans [70].

Laterality of CAN function has been an interesting finding of functional neuroimaging [20, 26, 51, 53, 58, 67, 71, 72]. Cortical structures that most consistently appear to display laterality in autonomic function are the amygdala, hippocampus, hypothalamus, and insula, though lateral specificity may vary between individuals [20]. Gender differences in the function of the CAN have similarly been revealed. Previous studies have revealed sex differences in HRV [73], implicating higher sympathetic tone in males compared to females. fMRI studies demonstrate gender differences in activation particularly in the insular cortex and dorsal ACC [71, 72, 74].

## Functional MRI of the central autonomic network in disease states

fMRI investigations of the CAN in healthy individuals have provided an excellent framework for understanding the vital central networks involved in ANS function. Further insight into the function of the CAN comes from fMRI studies in different disease states known to have abnormal ANS function. Though studies of CAN function or dysfunction in disease states are few, with often small numbers of subjects, they consistently implicate possible use of fMRI as a biomarker in these conditions, and demonstrate the need for further investigation in this area.

Neurologic conditions are often complicated by autonomic symptoms of variable severity. Sudden unexpected death in epilepsy (SUDEP) is the most common cause of premature death among people with epilepsy [75]. Seizure-induced autonomic (cardiac arrhythmia or hypotension) or respiratory (hypoventilation or apnea) dysfunction, or a fatal combination of these, have been postulated as likely causes. Of particular concern are epileptic seizures arising in, or rapidly propagating to, central autonomic control sites within the limbic system [76] resulting in damage to or dysregulation of critical autonomic structures. Cardiac alterations, particularly increased heart rate, are found more often in temporal lobe epilepsy (TLE) patients [77]. When compared with patients at low-risk for SUDEP, high-risk TLE patients exhibit reduced functional connectivity involving the bilateral brain stem, bilateral thalami, bilateral putamina, bilateral ACC, and left amygdala, [78], areas previously linked to increased SUDEP risk [79].

Alterations in CAN functional neuroimaging are also described in patients with neurodegenerative disorders. Many neurodegenerative conditions are associated with autonomic dysfunction, which may be severe and disabling in conditions such as Parkinson’s disease (PD) and multiple system atrophy (MSA). PD patients with higher burden of autonomic symptoms displayed significantly reduced functional connectivity between the hypothalamus and the striatum (caudate, putamen) and thalamus compared to PD patients with lower burden of these symptoms, suggesting that symptoms of autonomic dysfunction in PD are accompanied by alterations in CAN function, and its functional connections with the basal ganglia [80]. In analysis of task-free fMRI in patients with the behavioral variant of fronto-temporal dementia, the left anterior cingulate and left insular cortices stood out as the structures whose functional and structural integrity was most critical in maintaining cardiac vagal tone, implicating a left hemisphere-predominant contribution to maintaining parasympathetic outflow [81].

Increased fMRI signal was demonstrated in the hypothalamus in patients with short-lasting unilateral neuralgiform headache with conjunctival injection and tearing (SUNCT syndrome), a rare headache disorder that belongs to the trigeminal autonomic cephalgias (TAC) class of headache disorders [82]. Migraine patients were found to have increased functional connectivity between the hypothalamus and structures previously associated with sympathetic function (parahippocampal gyrus and cerebellar Crus I and II), as well as structures associated with parasympathetic function (the temporal pole, superior temporal gyrus, and cerebellar lobules V and VI), implicating altered hypothalamic connectivity as a central feature for autonomic symptoms in migraine patients [83].

Alterations in functional neuroimaging signals in the CAN are not unique to subjects with neurologic disease. fMRI studies in other disease states reveal functional alterations in autonomic regulatory areas in the brains of patients compared with controls. Heart failure (HF) patients are unable to adequately regulate sympathetic and parasympathetic output while at rest, or in response to body position, motor, or respiratory challenges which place demands on the autonomic nervous system [84, 85]. HF patients show decreased neural activation response to Valsalva in multiple autonomic and motor control areas, including the cerebellar cortices and vermis, hypothalamus, amygdala, left insula, ACC, left putamen, and bilateral postcentral gyri [86–88]. The fMRI responses in the cerebellum and insula in HF subjects were delayed or decreased in magnitude to the Valsalva challenge compared with controls [86]. Marked differences in timing, magnitude, and laterality of fMRI signals between HF patients and controls were also seen in the cerebellum [87]. Obstructive sleep apnea (OSA) subjects show significant amplitude and timing alterations in functional MRI signals in response to autonomic and respiratory challenges [89, 90]. They also demonstrate increased spontaneous MSNA-coupled fMRI activity alterations in the dorsolateral prefrontal, medial prefrontal, cingulate, precuneus and hippocampal-parahippocampal cortices [89, 90]. Additionally, altered MSNA-coupled fMRI signal intensity in the region of the medullary raphe nucleus, rostral VLM, and dorsolateral pons were identified [91]. Interestingly, continuous positive airway pressure (CPAP) treatment resulted in a significant reduction in resting MSNA levels and reversal of the previously identified brainstem MSNA-related functional brain changes back to control levels [92]. This finding warrants additional investigation in the use of fMRI as an outcome measure in the treatment of OSA.

Patients with congenital central hypoventilation syndrome (CCHS), a syndrome accompanied by severe disturbances in both ANS and respiratory function [93], show failure to achieve the appropriate magnitude and laterality of MRI signal response to an autonomic challenge in the amygdala and hippocampus [93]. CCHS patients also did not achieve appropriate heart rate changes with respiratory and autonomic challenges, which has been demonstrated in this condition previously. Interestingly, CCHS is another disorder of autonomic regulation associated with increased risk of sudden death [94].

## Conclusion and opportunities for future research

The advent of functional neuroimaging, particularly fMRI, has greatly advanced the state of understanding of the central nervous system role in the ANS in living humans. The scientific contributions of these technologies have been manifold. fMRI has allowed confirmation of the involvement of CAN structures in humans that were previously implicated in animal and lesional studies. It has also made possible the identification of CAN structures that had not been previously strongly linked with ANS activity. fMRI has greatly improved our understanding of the time course and order of central autonomic processing, as well as the interactions between autonomic structures. Crucially, the study of CAN dysfunction in disease states has profoundly expanded our understanding of physiologic autonomic changes in disease, and has opened the door for alternative interventions and clinical biomarkers for these conditions.

The use of fMRI in the study of the ANS is associated with several limitations, some of which are specific to this line of research while others are more general. First, while the standard spatial resolution of fMRI, which is usually 3.0–3.5 mm cubic voxels, but which can go down to around 1.5 mm [19], exceeds that offered by other neuroimaging modalities like EEG or PET, it may still be too limited in capturing task-associated or resting-state BOLD signals from smaller brain regions. While 3 T (3 T) MRI provides sufficient spatial resolution for supratentorial structures, infratentorial structures, specifically the brainstem which houses key components of the CAN, are more difficult to delineate reliably. This limitation may be overcome with more recent developments such as high-field and ultra-high-field MRI, which offer better spatial image acquisition capabilities, with the latter allowing scanning below 1.5 mm resolution. Similarly, there currently are very few MRI atlases available for brainstem anatomy, which often leads to the requirement of manual segmentation of these regions. This introduces a degree of variability between research groups.

Standard fMRI also may be limited by temporal resolution, driven to a large extent by the intrinsic properties of the hemodynamic response and a finite signal‐to‐noise ratio (SNR) [95]. Modern image acquisition protocols like multi-band MRI may allow acquisition of faster fMRI with more favorable SNR [96]. More generally, low SNR remains a major limitation in fMRI. This limitation is possibly amplified when studying several autonomic functions, where the signal and noise may originate from the same underlying source (for example, in respiratory control) [97]. State-of-the-art denoising techniques, and improvements in image acquisition are likely to continue and offer increasingly improved SNR in the coming years [98].

Inter-individual differences in fMRI results are routinely noted in studies [99]. These differences may be due to intrinsic differences between individuals, as is seen with structural MRI and anatomy and physiology in general [100], or may reflect early stages of disease. These differences may introduce higher levels of statistical variability, making it more difficult to interpret experimental results. It is important to stress that BOLD signal patterns and their interpretation are dependent on the task or outcome they are being associated with. BOLD signal in an individual is challenging to interpret and is constantly in flux. Much of the research using fMRI to understand the CAN has been limited by lack of uniformity in neuroimaging methodologies, inter-individual variation, and even variability in independent variable measures (HR, R–R interval, or HRV for example) [65]. Despite these limitations, there have been many consistencies in the results of these studies (Table 1), which increases confidence in the use of functional MRI in the study of the CAN.

Though fMRI provides a relatively high spatial and temporal resolution, many CAN structures are small and in areas that may be difficult to visualize, as discussed above. Ultra-high field MRI at 7 T (7 T) or above, may prove to be a valuable neuroimaging tool in the study of the CAN, allowing visualization and data acquisition from small structures and substructures of the brainstem and small areas abutting cerebrospinal fluid [101]. Very few functional studies of the CAN have been published using 7 T MRI to date [63, 101, 102]. Given the obvious strengths of 7 T MRI for use in studying the CAN, additional future research should be conducted using this modality.

Despite the plethora of studies using fMRI to uncover the normal functioning of the CAN, there have been surprisingly few studies investigating CAN dysfunction in disease states. In particular, much may be learned using fMRI to investigate CAN dysfunction in neurologic diseases such as the alpha-synucleinopathies, dementia states, epilepsy, and peripheral and autonomic neuropathies. Given the complex biophysical and physiological origins of the BOLD signal, which are still under active debate, and taking into account the non-trivial relationship between the BOLD signal and cerebral blood flow, caution is warranted in the interpretation of BOLD signal changes in both health and disease. Future research in this area should include both task-based and resting state designs, and careful selection of comparator populations.
